# Safety and Immunogenicity of Pertussis Vaccine Immunization during Pregnancy: A Meta-Analysis of Randomized Clinical Trials

**DOI:** 10.1155/2022/4857872

**Published:** 2022-12-21

**Authors:** Aidibai Simayi, Liguo Zhu, Hui Jin

**Affiliations:** ^1^Department of Epidemiology and Health Statistics, School of Public Health, Southeast University, No. 87 Dingjiaqiao, Nanjing, China; ^2^Key Laboratory of Environmental Medicine Engineering, Ministry of Education, School of Public Health, Southeast University, Nanjing, China; ^3^Department of Acute Infectious Disease Control and Prevention, Jiangsu Provincial Center for Disease Control and Prevention, Nanjing, China; ^4^National Health Commission (NHC) Key Laboratory of Enteric Pathogenic Microbiology, Jiangsu Provincial Center for Disease Control and Prevention, Nanjing, China

## Abstract

The objective of this meta-analysis is to assess the safety and immunogenicity of maternal pertussis vaccination based on randomized clinical trials. PubMed, Embase, Cochrane Library, Web of Science, China National Knowledge Internet, and Wan Fang Database were searched from inception up to the 8th of October 2021, using a protocol registered on PROSPERO with no. 42021287717, and a meta-analysis was conducted. We measured pooled geometric mean concentrations (GMCs) for IgG antibodies against pertussis and the incidence of serious adverse events (SAEs). We identified a total of 522 publications, and after a strict screening, we found that 6 RCTs were eligible for our meta-analysis. GMCs were determined with a standardized mean difference (SMD), and the pooled SMD of anti-PT, anti-FHA, and anti-PRN IgG from cord blood were 0.91 (95% CI: 0.58, 1.24), 1.03 (95% CI: (0.70, 1.35)), and 1.55(95% CI: 1.22, 1.88), respectively. The pooled OR of SAEs of women and infants did not show a statistical difference; the pooled ORs were 1.26 (95% CI: 0.78, 2.05); *P* = 0.35) and 0.61 (95% CI: 0.37, 1.01); *p* = 0.053), respectively. Infants of immunized women have significantly higher transplacental antibodies for protection against pertussis disease during the first 2 months of life.

## 1. Introduction

Pertussis is a highly contagious infectious respiratory disease, mainly caused by the bacterium *Bordetella* pertussis, and typically characterized by a prolonged cough [1]. Although pertussis is vaccine-preventable, it remains a global public health concern [2]. According to a recent report [3], it was estimated that there were 24.1 million cases of pertussis around the world in children aged <5 years with 160,700 deaths and many hospitalization admissions, some to pediatric intensive care units in 2014. Young infants < 6 months of age are at increased risk of pertussis-related complications, and infants <2 months of age are more likely to be under the threat of severe and potentially lethal complications [4]. Besides, pertussis continued to represent a serious public health problem in many countries, even in those with high rates of vaccination coverage [5].

Pertussis vaccination of pregnant women was first recommended in the United States (US) and the United Kingdom (UK) nearly a decade ago, in response to the resurgence of pertussis disease in the general population and multiple deaths in infants [6, 7]. The first routine pertussis vaccination occurs at six weeks to three months of age [8]. Infants under two months of age are the most vulnerable and have the highest rate of serious clinical complications requiring hospitalization and the highest mortality rate [9]. Meanwhile, maternal immunization is increasingly being recommended as a strategy to protect young infants from infectious diseases [10]. It was also reported that vaccination during pregnancy results in high levels of antibodies in the mother and the newborn. Furthermore, maternal tetanus, diphtheria, and acellular pertussis (Tdap) vaccination offers protection for neonates against clinical pertussis until primary vaccinations. The pertussis vaccine exists in both whole-cell (Tdwp) and acellular (Tdap) forms. The Tdap form has fewer adverse effects and seems to be as effective as the Tdwp formulation. As a result, the Tdwp preparation is only recommended when the Tdap form is not available [11]. Through transplacental transfer, antipertussis antibodies pass to the fetus, which is protected at the time of birth and during the first months of life [12]. However, performing clinical trials in pregnant women is challenging [13]; hence, the vast majority of immunogenicity and safety data has come from observational studies, which are prone to bias [14].

So far, several systematic reviews have investigated the effectiveness and/or safety of pertussis vaccination during pregnancy [15–20]. And yet none of them were specifically conducted as randomized clinical trials (RCT) or addressed the quantitative immune response comprising safety as well as immunogenicity for mother and child. Therefore, we performed a meta-analysis of RCTs to compare the immunogenicity and safety of pertussis vaccination during pregnancy.

## 2. Method

### 2.1. Data Sources and Search Strategy

This meta-analysis was conducted according to the Cochrane Collaboration guidelines and preferred reporting items for systematic reviews and meta-analyses protocols (PRISMA protocol) [21] and prospectively registered with PROSPERO (CRD-42021287717). We conducted a systematic search in electronic databases, including PubMed, Embase, Cochrane Library, Web of Science, China National Knowledge Internet, and Wan Fang Database, from inception up to the 8th of October, 2021, without language restrictions. The search strategy was built based on the following keywords and MeSH terms: “maternal,” “pregnancy,” “pregnant,” “pertussis,” “vaccination,” “vaccine,” “randomized controlled trials,” and filtered to “clinical trials” and “randomized controlled trials.”

### 2.2. Inclusion and Exclusion Criteria

Articles that met the following criteria were included: (a) RCTs; (b) primary studies; (c) the experimental group was treated with the Tdap vaccine during pregnancy; (d) control groups were treated with either a placebo, standard vaccination, or were unvaccinated; (e) reports at least one immunological response to vaccination. We excluded the following: (a) articles irrelevant to the topic; (b) duplicate publications; (c) trials of a cross-over study design; (d) animal and laboratory studies.

### 2.3. Quality Assessment

The methodological quality of each trial was evaluated for risk of bias using standard criteria: method of randomization; allocation concealment; patient, investigator, and outcome assessor blinding; selective outcome reporting; incomplete outcome ascertainment; and other potential sources of bias as recommended by the Cochrane Collaboration [22]. Each domain was categorized as low, high, or unclear.

### 2.4. Data Extraction

The data were carefully evaluated and extracted independently from all the eligible publications. The following data were collected from each study: (a) name of the first author, year of publication, and geographic setting; (b) study design; (c) type of vaccine during pregnancy; (d) study period; (e) the number of subjects in each group; (f) registration number of the trial; (g) gestational age in weeks of vaccination. To evaluate maternal pertussis vaccine immunogenicity, geometric mean concentrations (GMCs) for IgG antibodies against pertussis toxin (PT), filamentous haemagglutinin (FHA), and pertactin (PRN) in infants for all vaccine antigens were extracted from the trials. The following outcomes were considered for the meta-analysis: GMC after the infant series (at delivery, before primary vaccination, and after primary vaccination) of the Tdap vaccine. To evaluate safety, we measured the incidence of serious adverse events (SAEs) for women and their infants.

### 2.5. Statistical Analysis

The analysis of the immune response was performed mainly on cohorts according to protocol. Calculations of the GMCs of IgG antibodies against PT, FHA, and PRN were performed by taking the anti-ln of the means of the concentration transformations, and the GMCs were determined with the standardized mean difference (SMD). GMCs for antibodies against each vaccine component were calculated with 95% confidence intervals (CIs) in each study, and *p* values of less than 0.05 are significant. Statistical heterogeneity was assessed using the Cochran *Q* and *I*^2^ measures; an *I*^2^ value above 25% may be considered low heterogeneity, and a value above 50% and 75% were predefined as moderate and high heterogeneity, respectively [23]. Egger's test and Begg's test were conducted to explore the possibility of publication bias for the primary outcome [24, 25]. We also planned a priority to perform a leave-one-out sensitivity analysis to ascertain that the estimates were not driven by single trials. STATA, version 15.1 (StataCorp LP, College Station, TX, USA), was used for meta-analysis.

## 3. Result

### 3.1. Search Result

**Figure 1:**
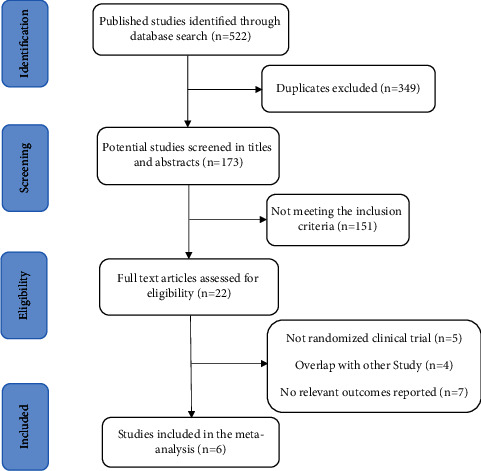
Flow chart of the literature searching process.

We identified a total of 522 publications, of which 349 were excluded due to duplication. Screening of titles and abstracts and inclusion criteria led to the exclusion of 151 publications. Of the remaining 22 studies, 6 were found to match our inclusion criteria (see flowchart in Figure 1).

### 3.2. Study Characteristics

Altogether, 6 randomized controlled trials were included in our final quantitative analysis [26–31]. Healthy pregnant women 18–45 years old who were not at known risk of pregnancy-related complications and had a normal singleton pregnancy were included in all these six studies. The total number of enrolled women was 709 in the experimental (Tdap) group and 691 in the control group. The basic information for the included studies is shown in Table 1. Participants were vaccinated either with the Tdap vaccine (experimental group) or with a placebo/TT/Td/unvaccinated (control group). The gestational age in weeks of vaccination was between 18 weeks and 36 weeks, the details of the RCTs identified in this study are shown in Table 1.

### 3.3. Quality Assessment

Most of the included studies had low biases, as shown by our quality assessment using the Cochrane assessment tool. The detailed quality assessment of each included study is shown in Supplementary File 1.

### 3.4. Meta-Analysis of Immunogenicity

**Figure 2:**
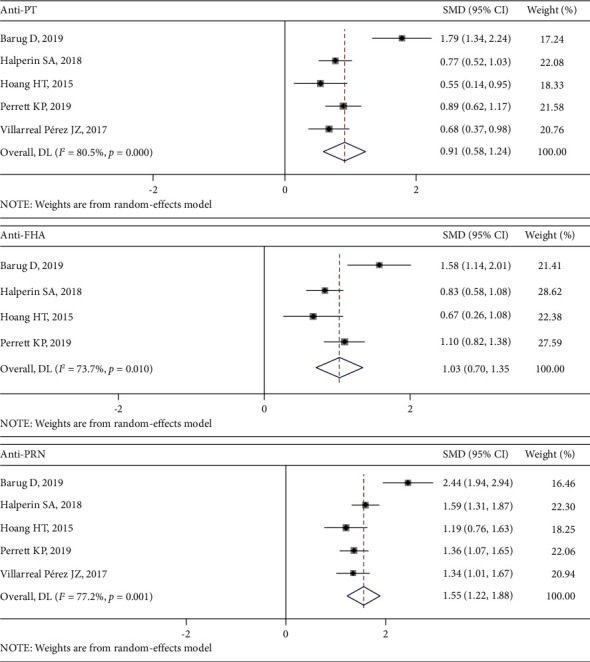
Forest plots of GMCs for pertussis antibodies in infants from cord blood.

Five studies were included in the analysis of anti-PT and anti-PRN IgG GMCs of infants from cord blood, and four studies reported the related GMCs for the FHA. The pooled SMD of anti-PT IgG from cord blood was 0.91 (95% CI: 0.58, 1.24; *P* < 0.0001). The pooled SMD of GMC for anti-FHA from cord blood was 1.03 (95% CI: 0.70, 1.35; *P* < 0.00001). Also, the pooled SMD of anti-PRN IgG from cord blood was 1.55 (95% CI: 1.22, 1.88; *P* < 0.00001). A random-effects model was employed due to the significant heterogeneity between different antibody responses among these studies (*I*^2^ = 80.5%, 73.7%, and 77.2%, respectively) (Figure 2). We removed the open-label trial of Barug et al. [26] for each analysis, and the heterogeneity of anti-PT and anti-PRN both sharply dropped to zero, and the *I*^2^ value of anti-FHA dropped to 41.5%, which fully explained that the high risk of bias was caused by the study design. The exclusion of this trial from the meta-analysis did not change the overall conclusion. Subsequently, we conducted meta-analyses according to different time points of Tdap vaccination (before and after the primary vaccination) of infants towards anti-PT, anti-FHA, and anti-PRN. Because the relevant antibodies in Perrett et al.'s study [30] were only measured in cord blood, they were not included in this part. The results are listed in Table 2, and related forest plots are shown in Supplementary File 2. The results suggest that GMCs for pertussis antibodies were higher in the Tdap group than those in the control group.

At delivery and before primary vaccination with a significant difference.After primary vaccination, there was significantly less anti-FHA antibody among the Tdap group when compared to the control group. However, no significant difference was noticed between anti-PT and anti-PRN antibodies. Sensitivity analyses showed that pooled SMDs did not change after removing any single study, indicating the stability of our results.

### 3.5. Meta-Analysis of Safety

**Figure 3:**
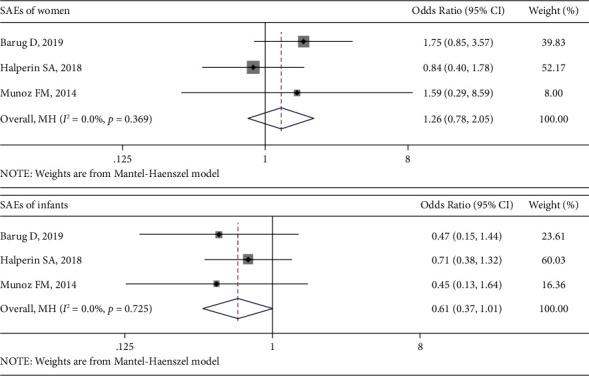
Forest plots of SAEs of women and infants.

Three articles [26, 27, 31] reported infant and pregnancy-/pregnant women-related SAEs. The pooled ORs of SAEs of women and their infants between the Tdap group and control group did not show a statistical difference; the ORs were 1.26 (95% CI: 0.78, 2.05; *P*=0.35; *I*^2^ = 0%) and 0.61 (95% CI: 0.37, 1.01; *P*=0.053; *I*^2^ = 0%), respectively. The forest plots are shown in Figure 3. For the sensitivity analysis of SAEs in women, removing the study with the highest weight (Halperin et al.) did not change the final result, pooled OR = 1.72 (95% CI: 0.89, 3.33; *P*=0.107; *I*^2^ = 0%).

### 3.6. Publication Bias

We used Begg's funnel plot and Egger's test to assess the possible publication bias of the included studies. All *P* values of Egger's test and Begg's test were >0.05, and visual inspection of Begg's funnel plots did not suggest evidence of publication bias. Relevant Begg's plots and Egger's plots are shown in Supplementary File 3.

## 4. Discussion

To the best of our knowledge, this is the first meta-analysis exploring the immunogenicity and safety of maternal pertussis vaccination based on RCTs. This meta-analysis synthesized evidence about the immunogenicity and safety of Tdap vaccination during pregnancy in 6 studies involving more than 1400 pregnant women and infants. We used a systematic strategy and broad search terms in multiple databases to identify as many published clinical trials as possible. Maternal pertussis immunization has undergone a paradigm shift in recent years as evidence emerges of robust effectiveness and safety in protecting young infants and their mothers against pertussis [32]. GMCs against pertussis were assessed by performing an ln transformation, to get a more intuitional understanding of the immunogenicity of vaccines.

For immunogenicity, our results from the analysis of 6 RCTs suggested that GMCs of anti-PT, anti-FHA, and anti-PRN were higher in the Tdap group than the control group at delivery and before primary vaccination of infants, which is consistent with the included studies [26–31]. However, after primary vaccination, anti-PT and anti-PRN did not show statistical differences between the Tdap group and the control group, and GMCs of anti-FHA were statistically less in the Tdap group than the control group, suggesting that maternal immunization with Tdap resulted in high concentrations of pertussis antibodies in infants during the first 2 months of life until they get primary vaccinated. This supports the recommendation of Tdap vaccination during pregnancy to prevent early-infant pertussis disease.

For safety, significant differences were demonstrated in the comparisons of the incidence of serious SAEs, which mainly included pregnancy-induced hypertension, pancreatitis, acute appendicitis, fetal distress resulting in a *C*-section, congestive heart failure, and gastroenteritis. Both SAEs of pregnant women and their infants showed no significant differences. According to the included studies, none of the SAEs in women and their infants were judged to be attributable to the Tdap vaccine, except that four of these pregnancy-related SAEs were assessed as possible vaccine-related (preeclampsia, premature delivery, and HELLP syndrome (hemolysis, elevated liver enzymes, low platelet count) in 1 Td recipient and gestational hypertension in 1 Tdap recipient) [31]. Hoang et al.'s study [28] reported 7 SAEs but did not reveal the distribution of the incidences, so we did not include this in the meta-analysis. Other included studies also reported the incidence of non-SAEs [26, 28, 30], mainly redness and mild local pain, but they were either without significant differences between the Tdap group and control group or without eligible data for pooled analysis; therefore, we did not perform a meta-analysis about non-SAEs. Overall, our results of the Tdap vaccine's safety are consistent with the included studies.

Infants are specifically prone to bradycardia, hypotension, and cardiac arrest from pertussis. The development of pulmonary hypertension has been increasingly recognized as a factor contributing to infant mortality at an early age, as it may lead to worsening systemic hypotension and hypoxia [33]. Some of the included studies [27, 30, 31] reported the occurrence of obstetric or fetal complications in the Tdap and control groups; however, there were no significant differences between them, and the reported data were not eligible for meta-analysis; thus, we did not perform meta-analysis based on obstetric or fetal complications.

Several limitations of the present study must be acknowledged. First, we searched only six databases, and some unpublished studies or publications in other databases may not have been identified. Second, only a limited number of published RCTs directly compare the immunogenicity and safety of Tdap maternal vaccination. While RCTs are desirable for addressing the impacts of antenatal vaccination timing on vaccine immunogenicity, there are limitations on study design due to the ethical issues raised by delaying vaccination. Third, there was significant heterogeneity among the studies that evaluated GMCs of pertussis antibodies in cord blood and after primary vaccination. The result of the sensitivity analyses that were performed indicated that the possible reason for the heterogeneity was a different trial design; however, the overall conclusions were not changed. Fourth, the infants in the included study did not get the postvaccine at the same age, with a one-month delay [26]; hence, the corresponding results for GMCs might include bias. Additionally, the doses of the Tdap vaccine have slight differences among each included study, and the intervention of the control group was different as well, which may influence the final result. Finally, findings from the studies included in this paper are not necessarily applicable to infants and children globally. Follow-up periods were often of necessity short, mostly to less than 12 months of age. Most of the studies were conducted in communities with many years of use of pertussis vaccines.

## 5. Conclusion

This meta-analysis shows significant evidence that infants of immunized women had significantly higher transplacental antibodies for protection against pertussis disease during the vulnerable newborn period before they received their primary immunizations. We analyzed the incidences of SAEs in women and infants as well, and our results support the recommendation for routine Tdap immunization in pregnancy to improve the protection of infants against pertussis disease before primary infant immunization.

## Figures and Tables

**Table 1:** Description of the characteristics of the included studies.

Source	Country	Type of vaccines during pregnancy (experimental group/control group)	Study period	Study design	Registration no.	Gestational age in weeks of vaccination	Participants no. (experimental group/control group)
Barug et al. [26]	Netherlands	Tdap/---	January 2014–March 2016	RCT, open-label	EudraCT 2012-004006-9/NTR number NTR4314	30–32 weeks	58/60
Perrett et al. [30]	Australia, Canada, Czech Republic, Finland, Italy and Spain	Tdap/placebo	October 2015–October 2017	RCT, observer-blind	NCT02377349	27–36 weeks	341/346
Munoz et al. [27]	The United States	Tdap/placebo	October 2008–May 2012	RCT, double-blind	NCT00707148	30–32 weeks	33/15
Hoang et al. [28]	Vietnam	Tdap/TT	NA	RCT	NA	18–36 weeks	52/51
Villarreal Pérez et al. [29]	Mexico	Tdap/placebo	September 2011–August 2014	RCT, double-blind	NCT01445743	30–32 weeks	90/81
Halperin et al. [31]	Canada	Tdap/Td	November 2007–June 2011	RCT, observer-blind	NCT00553228	≥30 weeks	135/138

*Note.* “---” = not received Tdap or placebo during pregnancy. TT = tetanus toxoid. Td = tetanus and diphtheria toxoids. NA = not applicable.

**Table 2:** Meta-analysis results of GMCs for pertussis antibodies before and after primary vaccination of infants.

	Study number	Pooled SMD (95% CI)	*P* value	*I* ^2^ (%)	Effect model
*Before primary vaccination*					
Anti-PT	4	0.75 (0.28, 1.22)	0.002	86.3	Random
Anti-FHA	3	0.90 (0.41, 1.39)	<0.0001	80.9	Random
Anti-PRN	4	1.37 (0.90, 1.83)	<0.0001	84.0	Random

*After primary vaccination*					
Anti-PT	4	−0.016 (−0.32, −0.01)	0.059	0	Fixed
Anti-FHA	3	−0.20 (−0.39, −0.01)	0.039	10.8	Fixed
Anti-PRN	4	−0.05 (−0.50, 0.40)	0.819	85.7	Random

## Data Availability

Data are included within the article.

## Supplementary Materials

Supplementary Materials Supplementary File 1. Table S1. Risk of bias assessment of each included study. Supplementary File 2. Supplementary figure 3. Begg's funnel plots and Egger's plots. Supplementary File 3. Figure S2. Forest plots of GMCs of pertussis antibodies before and after primary vaccination.
