# Supplementary material for: Safety and Immunogenicity of Pertussis Vaccine Immunization during Pregnancy: A Meta-Analysis of Randomized Clinical Trials
Source: J Trop Med. 2022 Dec 21;2022:4857872. doi: 10.1155/2022/4857872 (PMC9797314; doi:10.1155/2022/4857872)
Supplement: Supplementary Materials — Supplementary File 1. Table S1. Risk of bias assessment of each included study. Supplementary File 2. Supplementary figure 3. Begg's funnel plots and Egger's plots. Supplementary File 3. Figure S2. Forest plots of GMCs of pertussis antibodies before and after primary vaccination. [file 4857872.f1.zip › Supplementary File1.docx]

Table S1. Risk of bias assessment of each included study ^a^

| Study Validity  Domains | Sequence generation | Allocation Concealment | Blinding of participants and personnel and outcome assessors | Incomplete outcome data | Selective outcome reporting | Other sources  of bias |
| --- | --- | --- | --- | --- | --- | --- |
| **Probiotics** |  |  |  |  |  |  |
| Barug D, 2019 | Low | Low | High^c^ | Low | Low | Low |
| Halperin SA, 2018 | Low | Low | Low | Low | Low | Low |
| Hoang HT, 2015 | Low | Unclear^b^ | Unclear^b^ | Low | Low | Low |
| Munoz FM, 2014 | Low | Low | Low | Low | Low | Low |
| Perrett KP, 2019 | Low | Low | Low | Low | Low | Low |
| Villarreal Pérez JZ, 2017 | Low | Low | Low | Low | Low | Low |

a. Each domain has been evaluated as being “High”, “Low”, or “Unclear” regarding the risk of bias following the guidelines of Cochrane Collaboration’s tool for assessing risk of bias “Low” in all Domains would place a study at “Low Risk of Bias”; “High” in any of the Domains would place a study at “High Risk of Bias”; “Unclear” in any of the domains would place the study at “Unclear Risk of Bias”.

b. Not mentioned

c. open-labeled
