# Supplementary material for: Safety and Immunogenicity of Pertussis Vaccine Immunization during Pregnancy: A Meta-Analysis of Randomized Clinical Trials
Source: J Trop Med. 2022 Dec 21;2022:4857872. doi: 10.1155/2022/4857872 (PMC9797314; doi:10.1155/2022/4857872)

**Figure S2. forest plots of GMCs of pertussis antibody before and after primary vaccination**

Before primary vaccination

Anti-PT


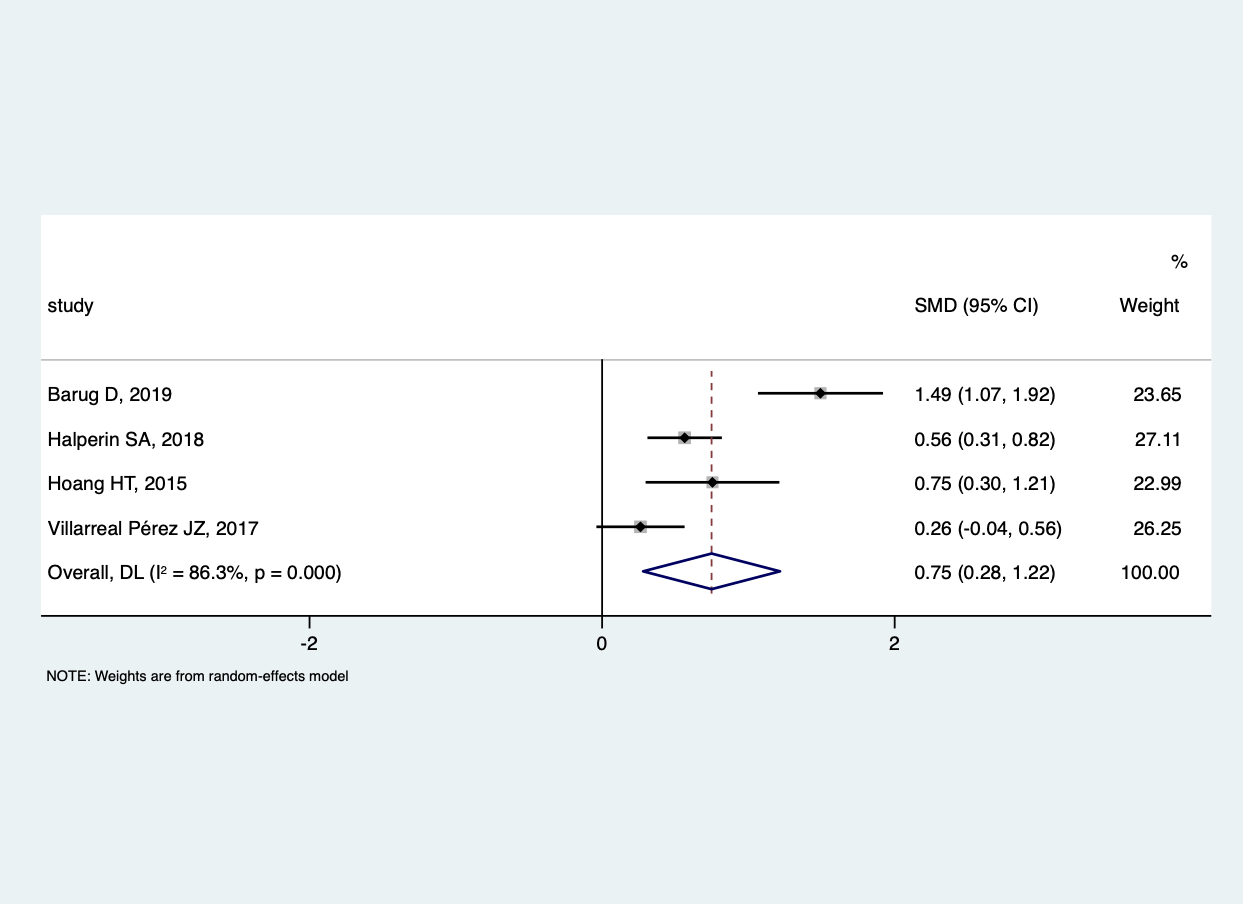


Anti-FHA


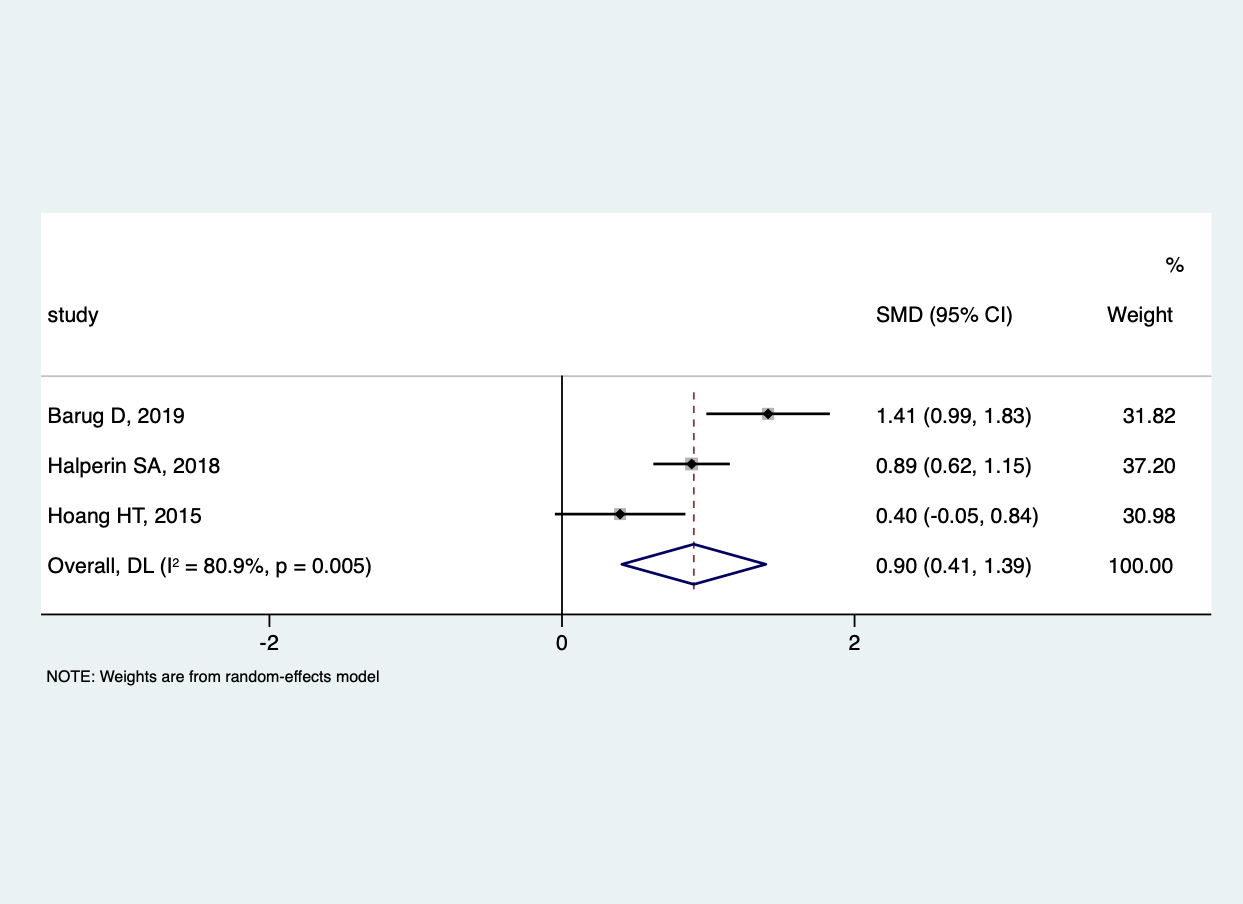


Anti-PRN


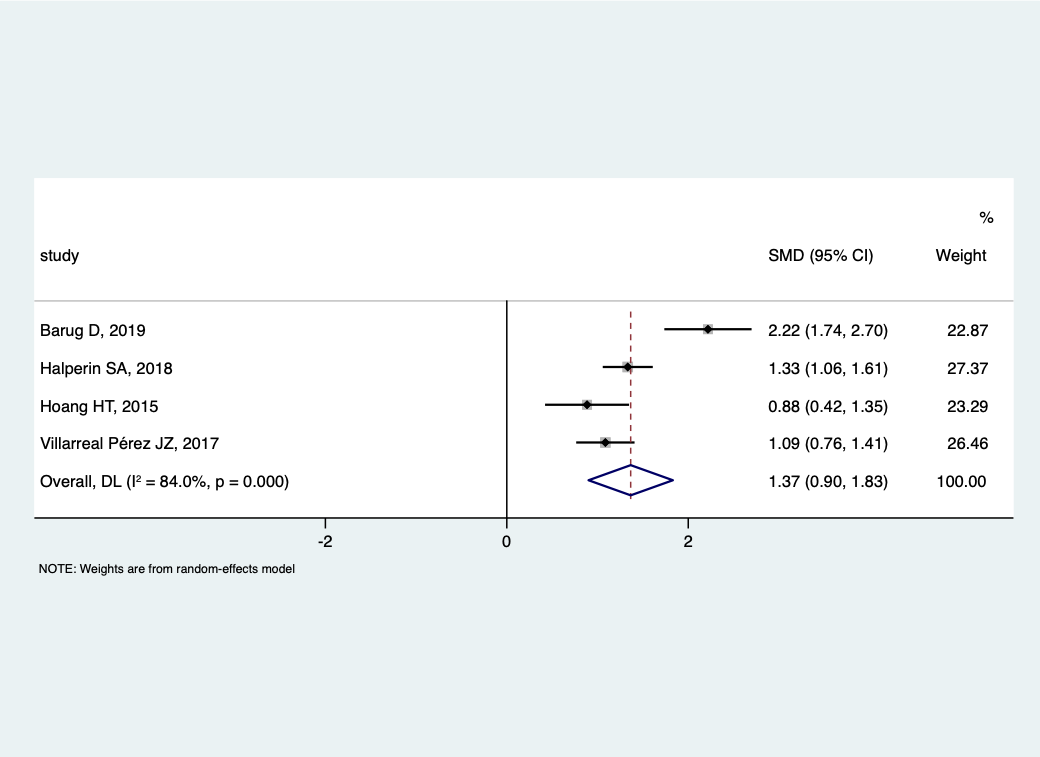


After primary vaccination

Anti-PT


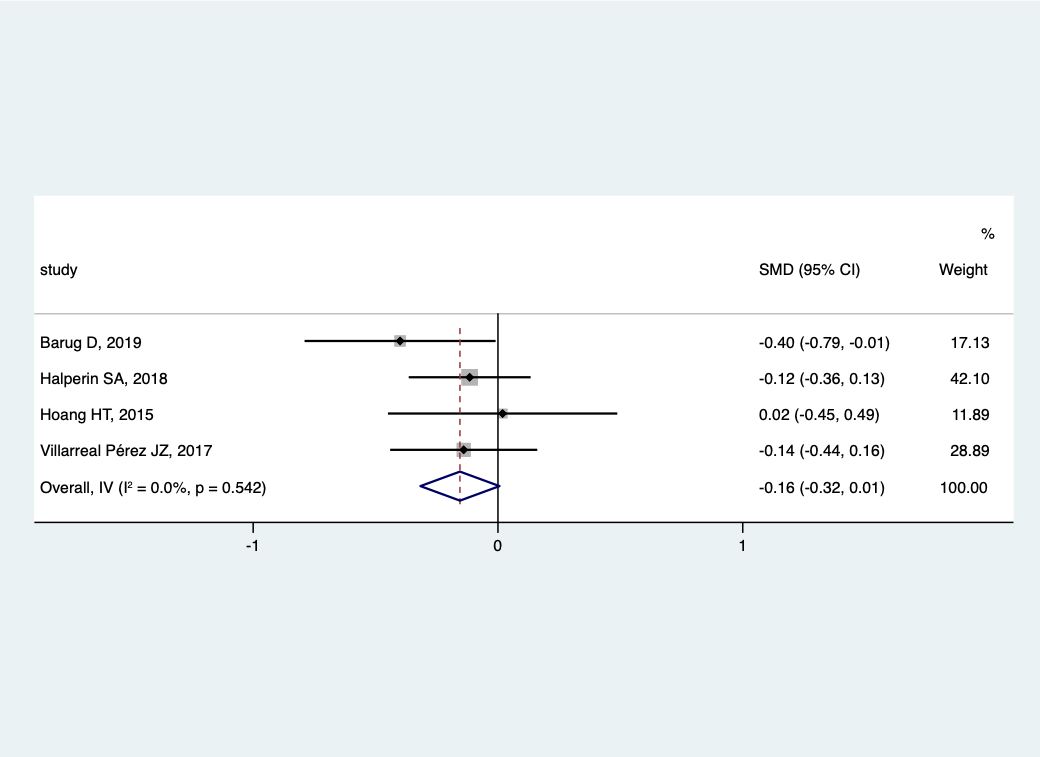


Anti-FHA


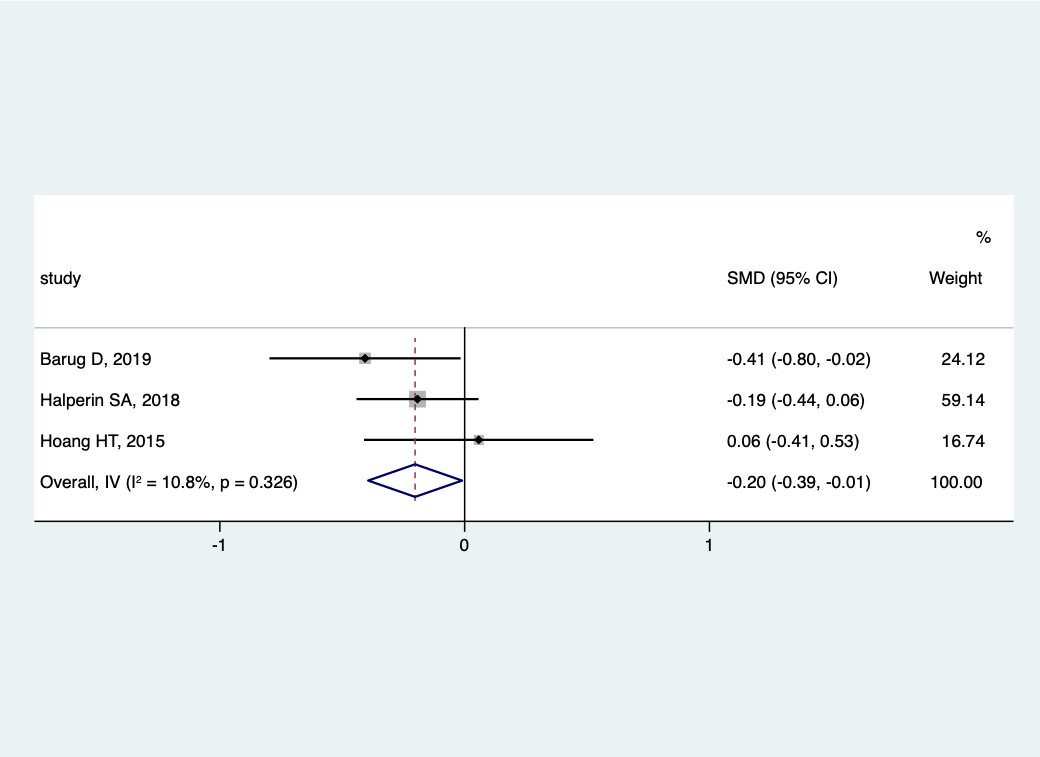


Anti-PRN


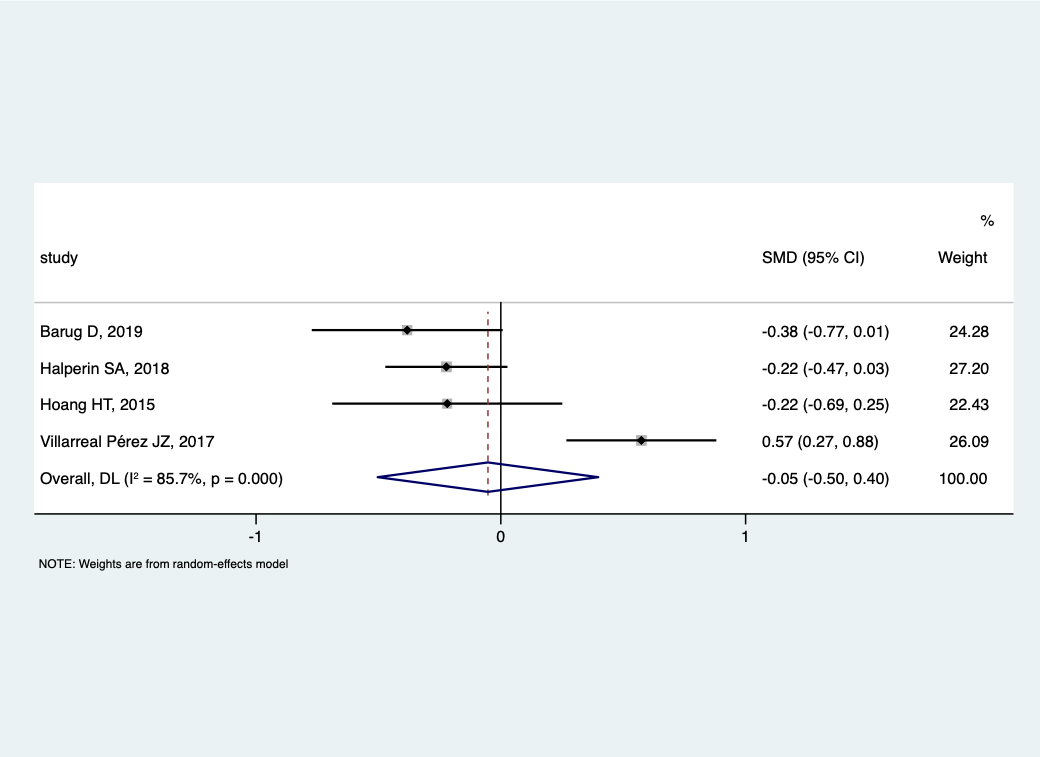

Supplement: Supplementary Materials — Supplementary File 1. Table S1. Risk of bias assessment of each included study. Supplementary File 2. Supplementary figure 3. Begg's funnel plots and Egger's plots. Supplementary File 3. Figure S2. Forest plots of GMCs of pertussis antibodies before and after primary vaccination. [file 4857872.f1.zip › Supplementary File2 (1).docx]
