# Supplementary material for: Safety and Immunogenicity of Pertussis Vaccine Immunization during Pregnancy: A Meta-Analysis of Randomized Clinical Trials
Source: J Trop Med. 2022 Dec 21;2022:4857872. doi: 10.1155/2022/4857872 (PMC9797314; doi:10.1155/2022/4857872)

**Supplementary Fig3. Begg’s funnel plots and Egger’s plots**

Cord blood

Anti-PT


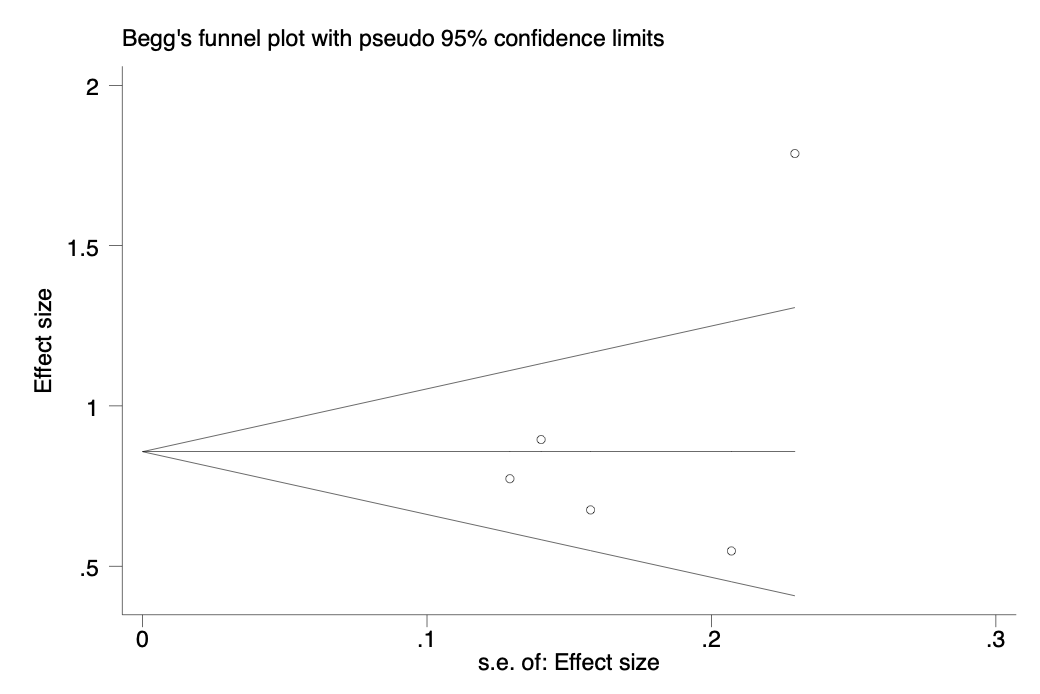


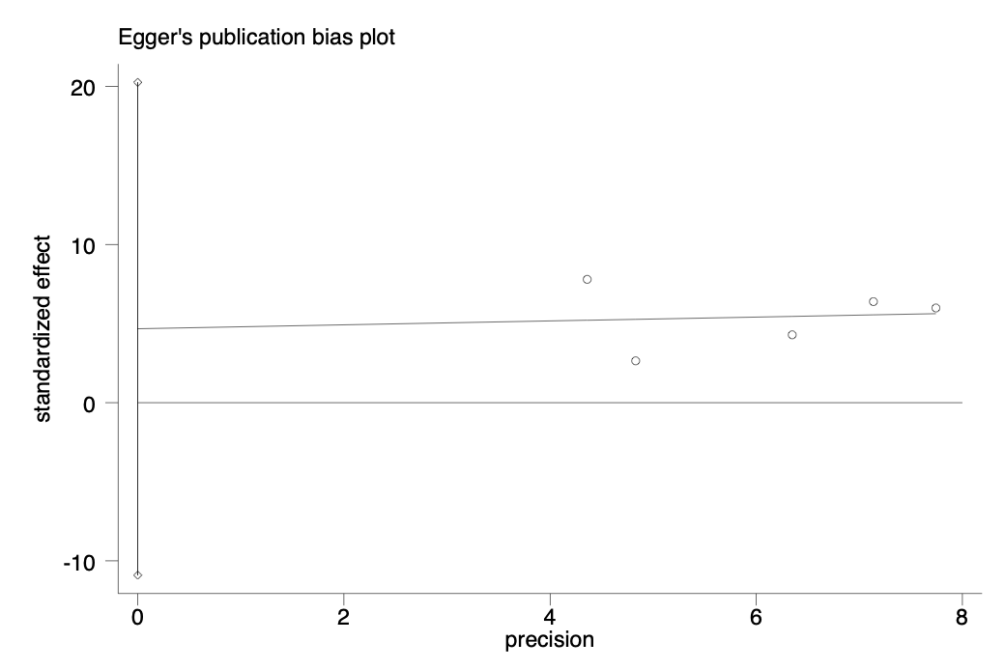


Anti-FHA


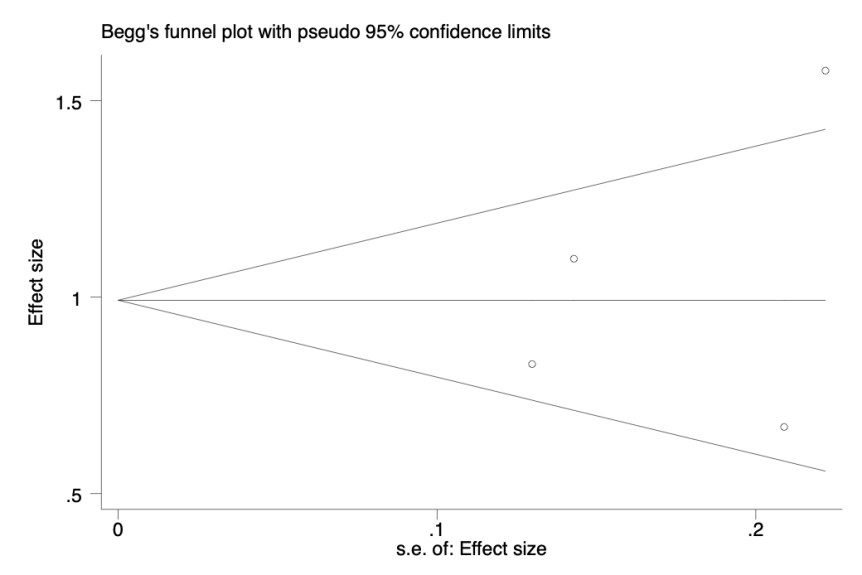


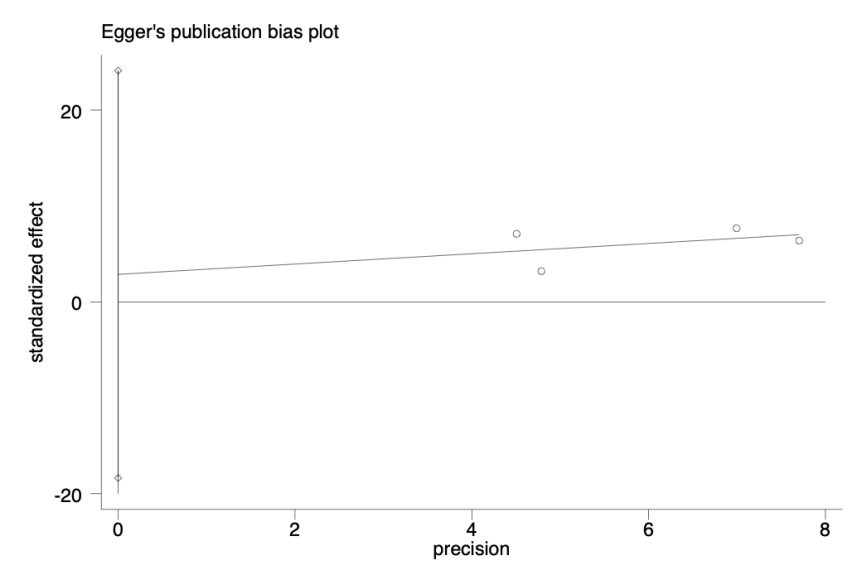


Anti-PRN


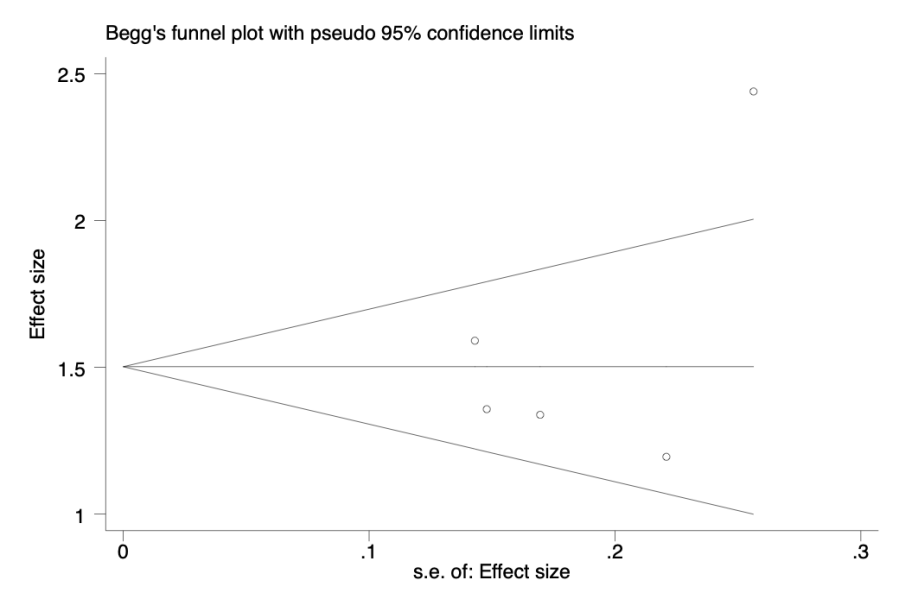


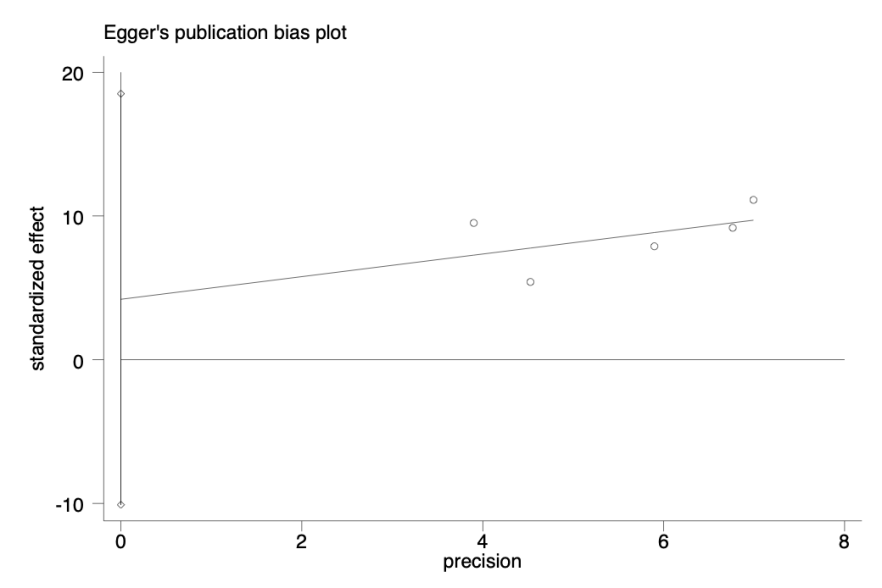


Before primary vaccination

Anti-PT


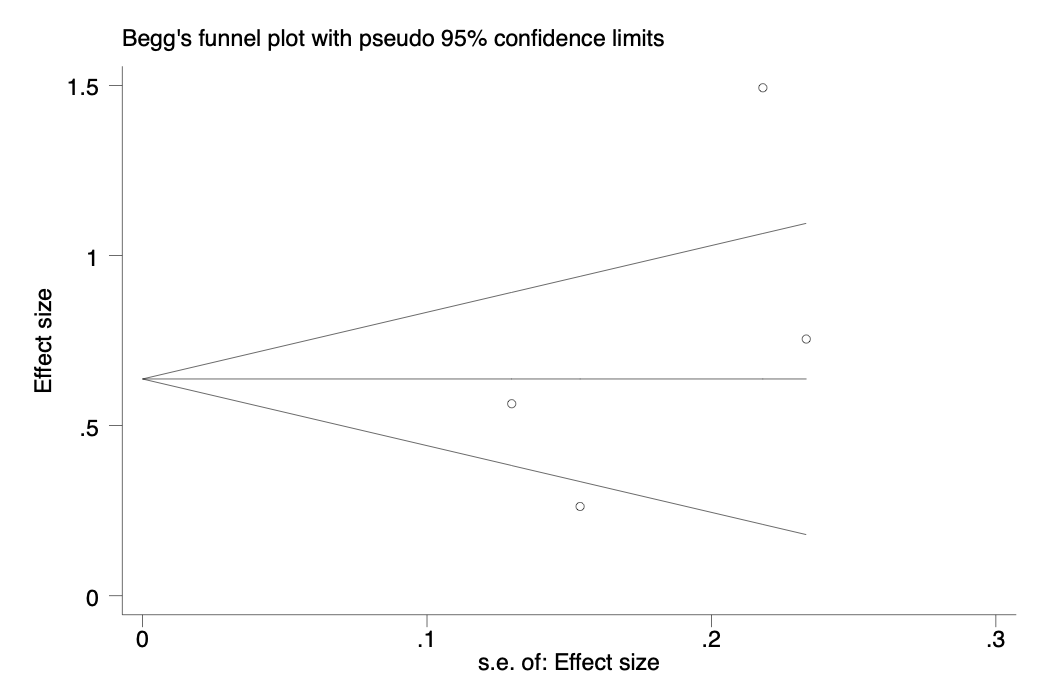


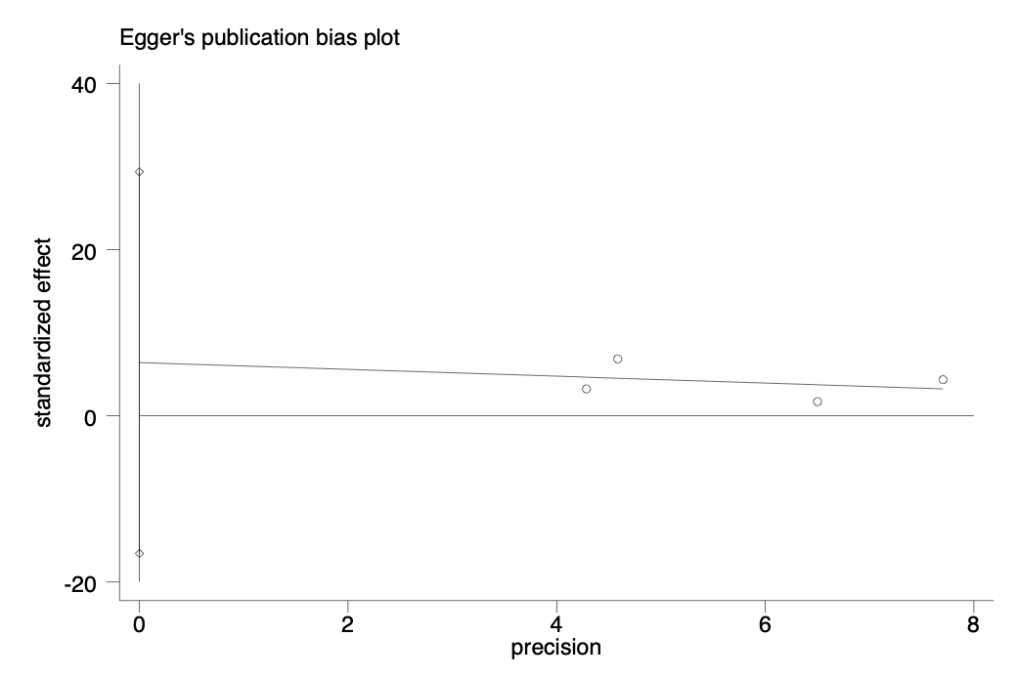


Anti-FHA


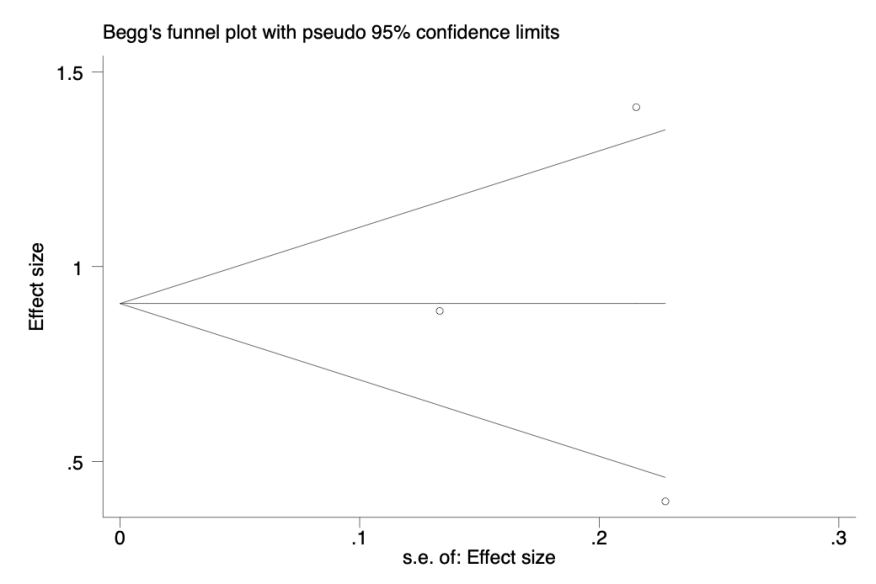


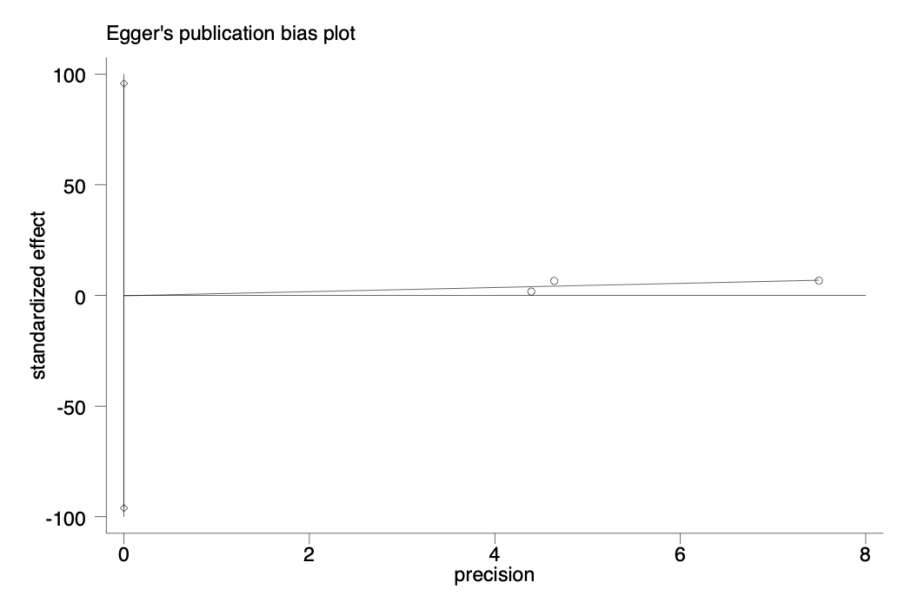


Anti-PRN


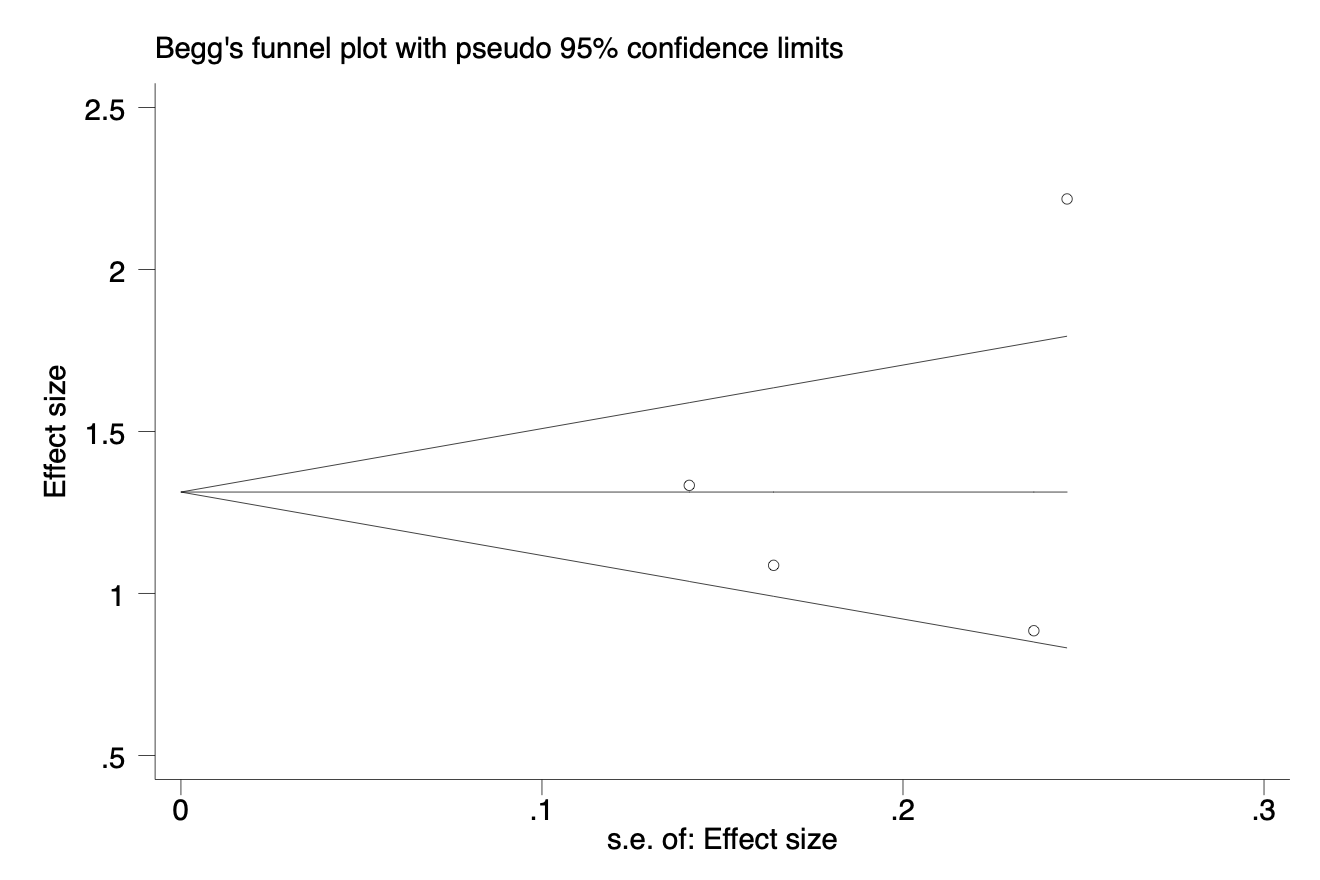


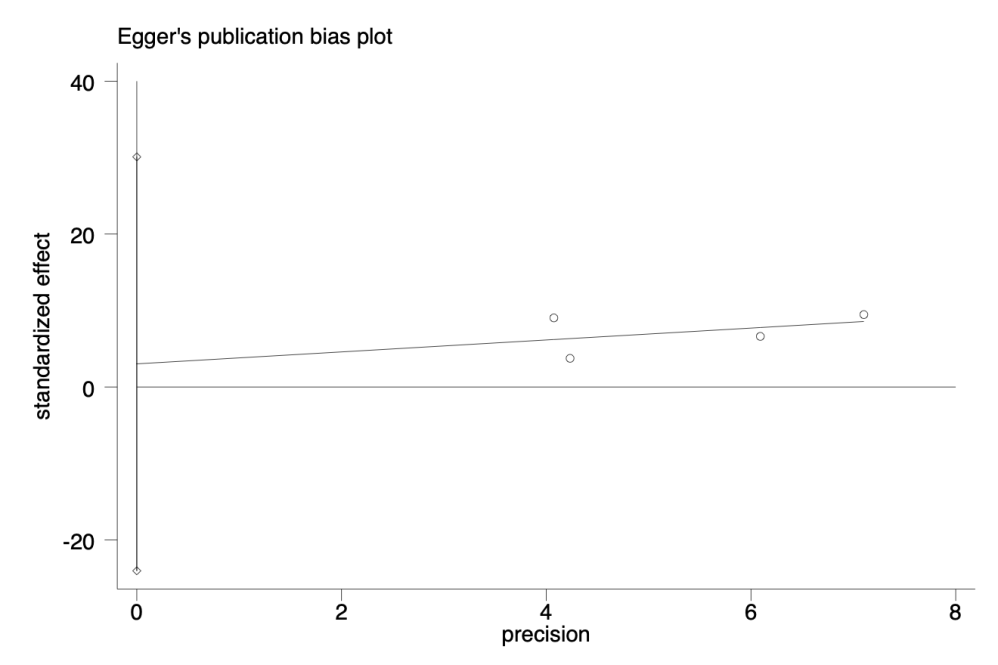


After primary vaccination

Anti-PT


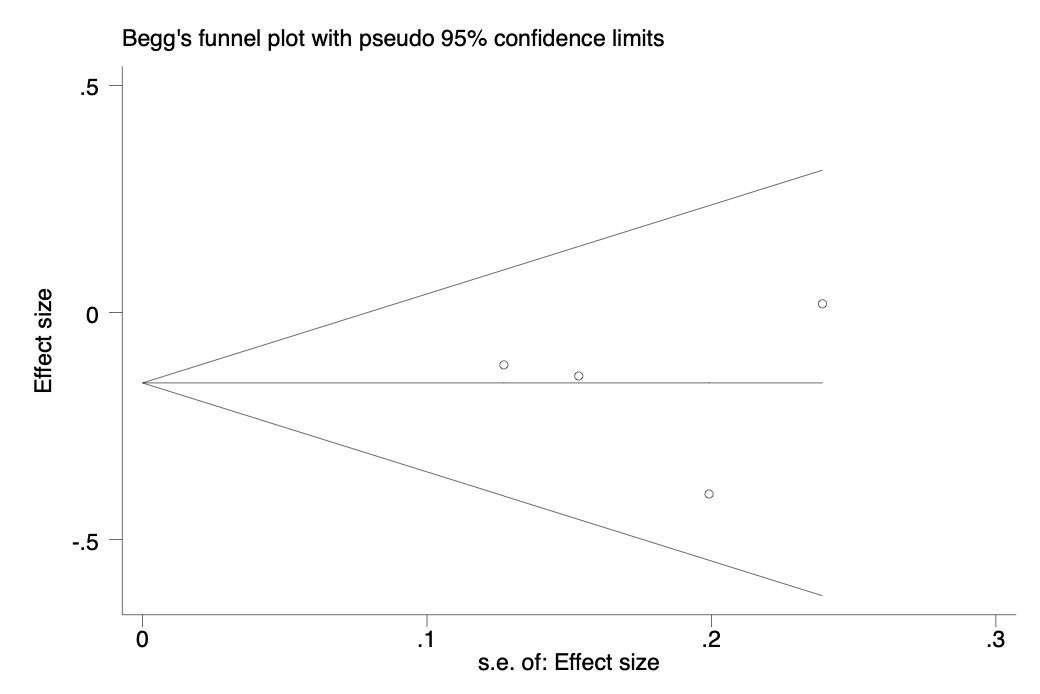


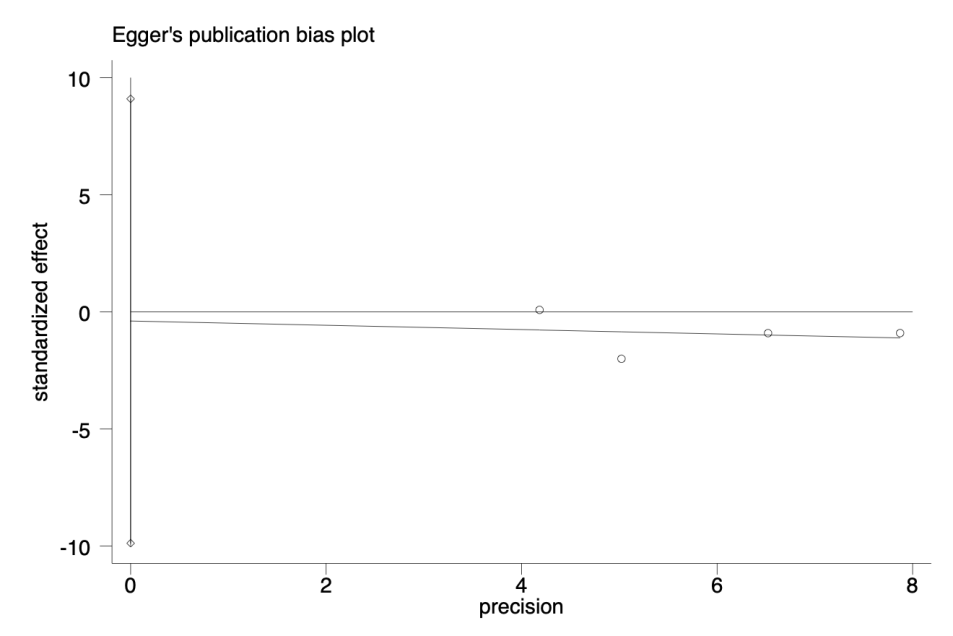


Anti-FHA


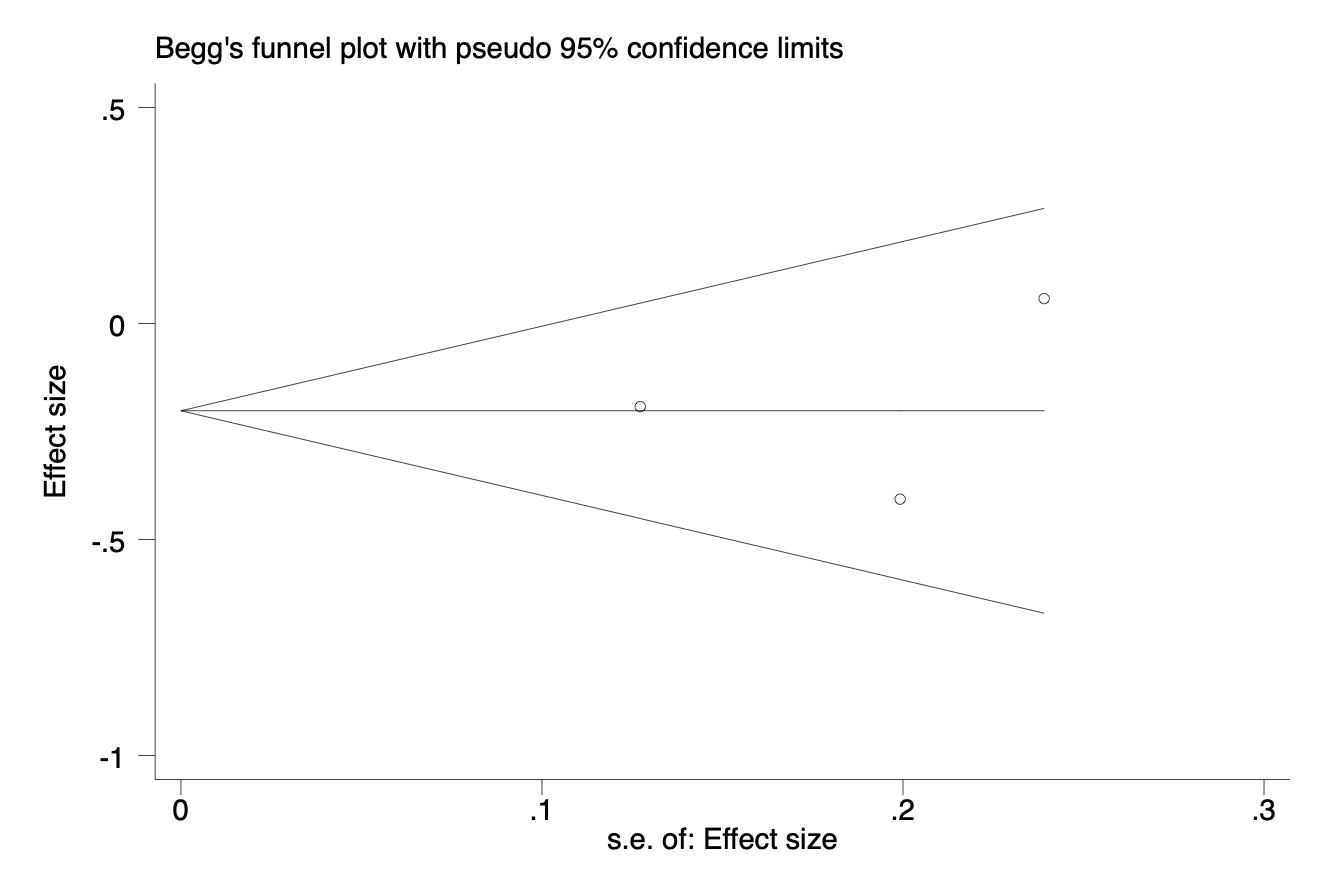


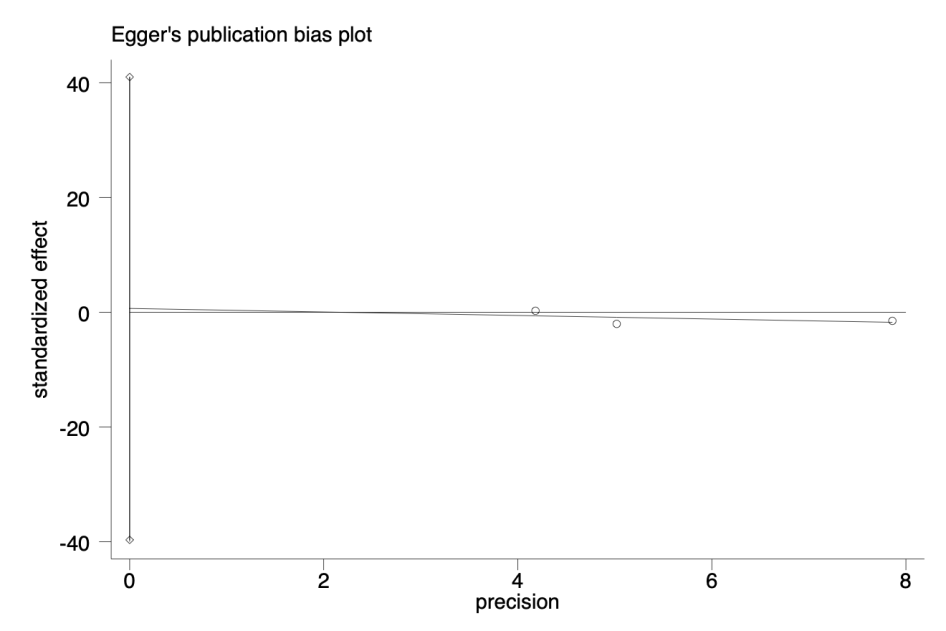


Anti-PRN


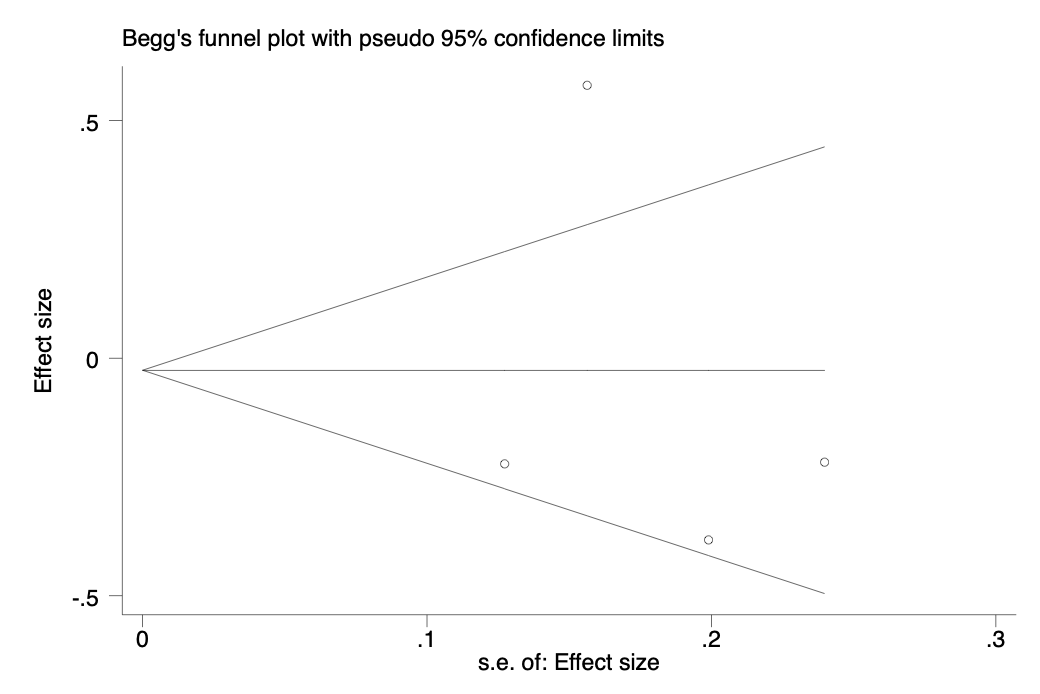


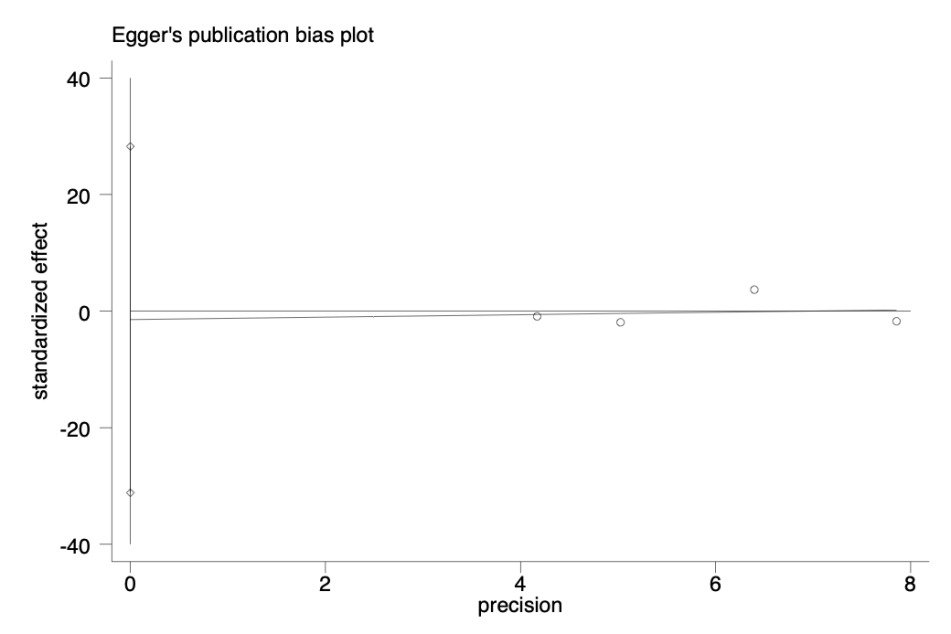


SAEs in women


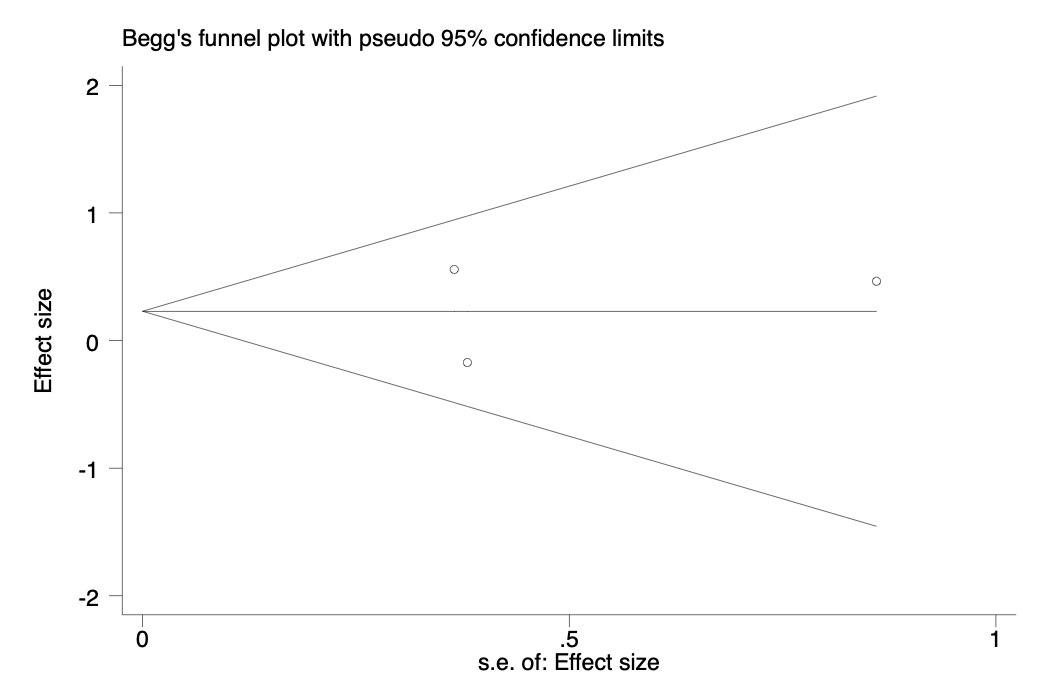


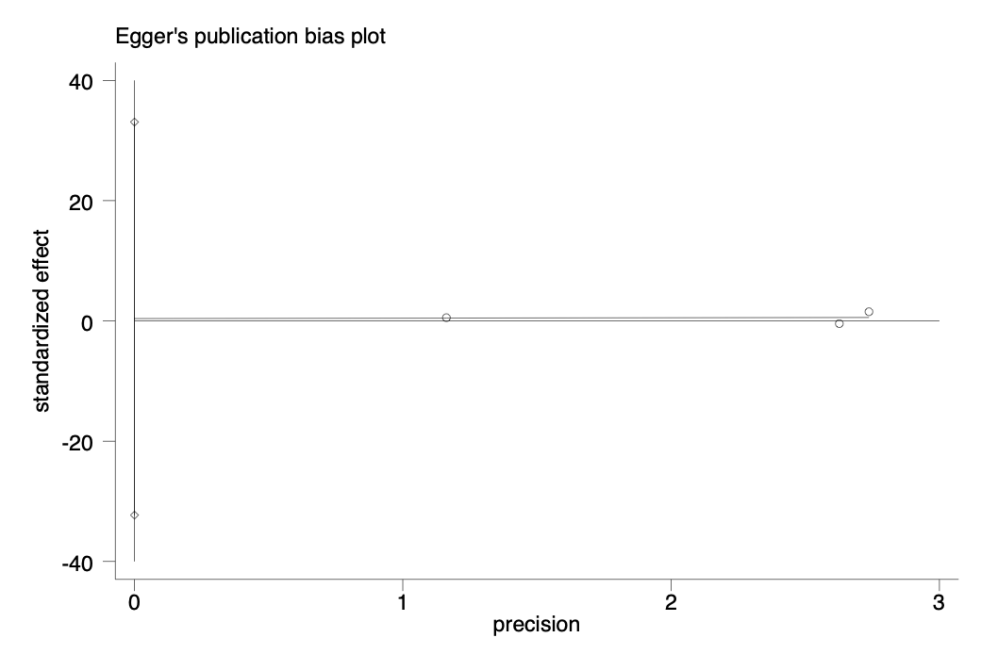


SAEs in infants


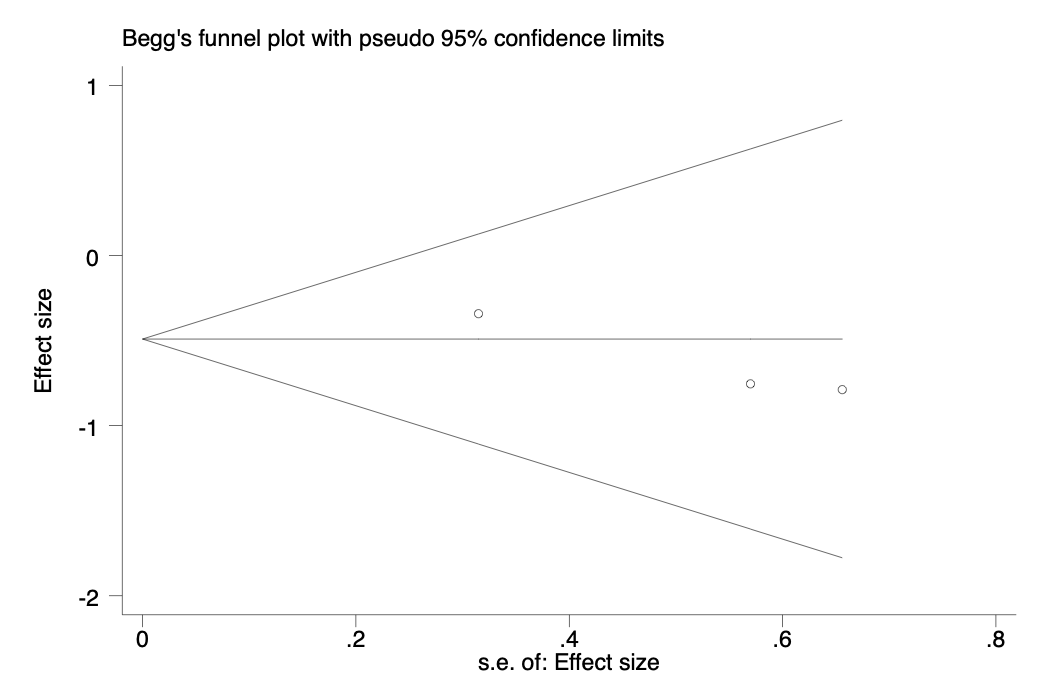


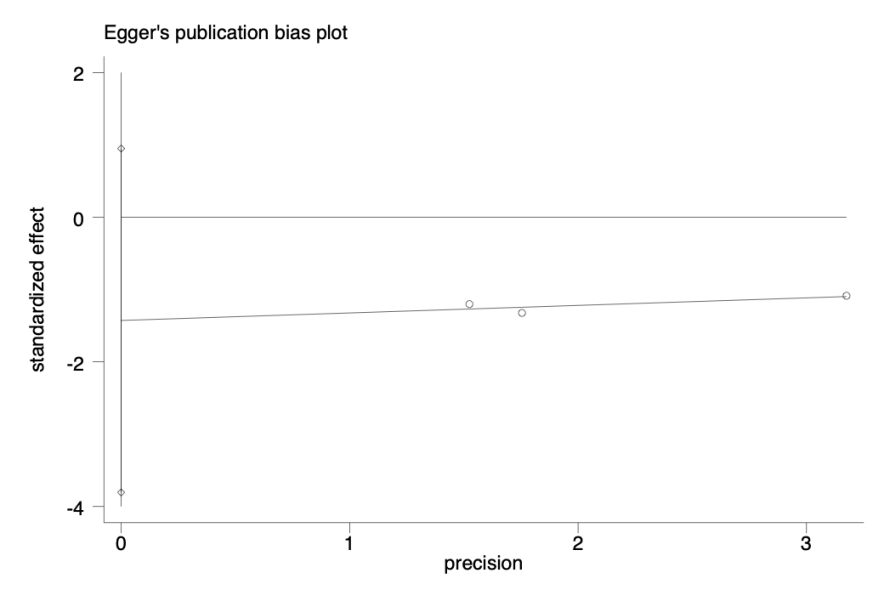

Supplement: Supplementary Materials — Supplementary File 1. Table S1. Risk of bias assessment of each included study. Supplementary File 2. Supplementary figure 3. Begg's funnel plots and Egger's plots. Supplementary File 3. Figure S2. Forest plots of GMCs of pertussis antibodies before and after primary vaccination. [file 4857872.f1.zip › Supplementary File3 (1).docx]
